# Emissions of pesticides in the European Union: a new regional-level dataset

**DOI:** 10.1038/s41597-023-02753-4

**Published:** 2023-12-05

**Authors:** Angel Udias, Francesco Galimberti, Chiara Dorati, Alberto Pistocchi

**Affiliations:** 1 European Commission Joint Research Centre, Ispra, Italy; 2 Universidad Rey Juan Carlos, Madrid, Spain; 3 ICPS International Centre for Pesticides and Health Risk Prevention, Milan, Italy

**Keywords:** Environmental impact, Sustainability, Agriculture

## Abstract

We present a European Union (EU)-wide dataset of estimated quantities of active substances of plant protection product applied on crops (also called “emissions”). Our estimates are derived from data reported by eight EU countries and extrapolated to encompass all EU regions using regression models. These models consider both climate and agricultural land use data. This allows us to spatially represent pesticide use at NUTS Level 3 of the European statistical mapping units, and within various agricultural land cover classes in each region. We compare our estimates with aggregated data provided by EUROSTAT and with independent, detailed data for the United Kingdom, highlighting an error typically within one order of magnitude. Our estimates can provide insights into the distribution and patterns of pesticide use in the EU around the year 2015. The estimate is most reliable for Western and Southern Europe. Outside these regions, data scarcity makes extrapolation more uncertain, potentially limiting the ability to accurate depict regional variations in pesticide use.

## Background & Summary

Plant protection products (PPPs) may have significant impacts on ecosystems, biodiversity and human health^1,2^. However, a comprehensive risk assessment of the large number of pesticides currently in use remains challenging^3,4^.

One of the main hurdles in studying the spatial patterns of PPP pollution and cumulative toxicity is the lack of data on the use of PPP active substances (AS)^5–7^. At the European Union (EU) scale, this information has only been available in a highly aggregated form (total national sales of relatively broad groups of AS) due to confidentiality restrictions^8,9^. Access to reliable, granular data on PPP use is crucial to inform policies and increase transparency^10,11^.

As a surrogate of the accurate and granular data so far unavailable, we produce an estimation of PPP AS use across the EU around the year 2015, by extrapolating the relatively detailed data available in some countries. Specifically, we have collected national data on the use or sales of 309 individual active substances from eight EU Member States (Belgium, Denmark, France, Germany, Ireland, Italy, The Netherlands, Spain: see^12^. By applying multiple linear regression models, we predict the annual amount of 152 PPP AS used in agriculture across the EU. Cross-validation with available data indicates discrepancies of less than one order of magnitude. Maps of the estimated use of PPP AS have been developed for different assessment purposes, including the calculation of loads to and toxicity in the EU river network as presented in^13^.

## Methods

The amount of PPP AS present in the environment is related to the geographic extent of application, the doses applied, the persistence of the substances and that of their metabolites^14^.

We aim at estimating emissions of PPP AS within spatial units corresponding to small regions corresponding to level 3 of the European Nomenclature of Territorial Units for Statistics, NUTS3 (https://ec.europa.eu/eurostat/web/nuts/background). To accomplish this, we independently estimate the emissions of each plant protection product active substance (PPP AS) specific to each of the six distinct land cover classes (arable land, fruit tree, grassland, olive groves, vineyards, rice fields). The aggregate emissions estimate for each spatial unit at NUTS3 level is obtained by summing the projected emissions in these six classes.

**Fig. 1:**
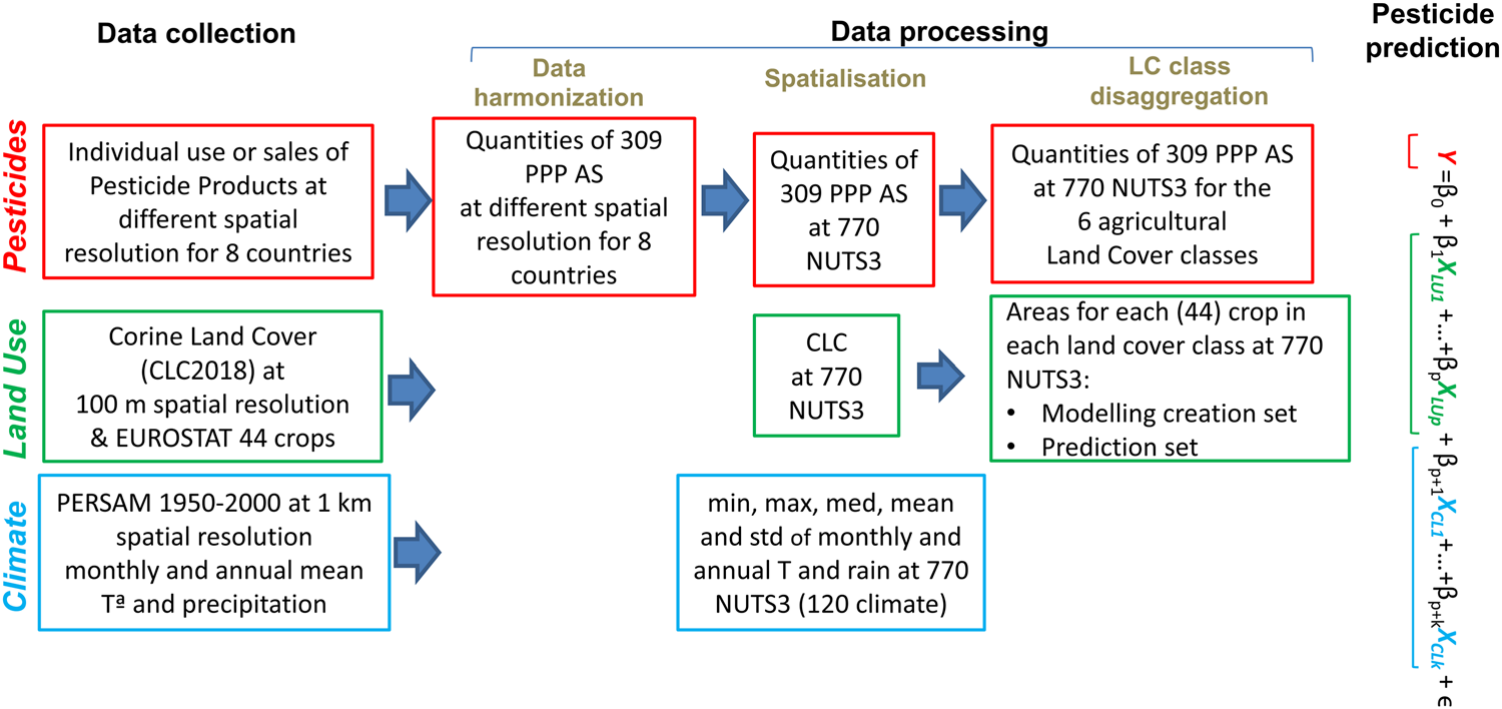
General scheme of the data collection, data processing and pesticide prediction steps. The explanatory variables of the prediction model are the land use (in green) and climate (in blue) variables, while the response variables are the data related to the PPP AS quantities (in red).

We first collected data on use of the various PPP AS in selected countries. Each PPP AS is associated with the crops where application can be expected. We apportion the total use to individual regions within the country on the basis of the crops grown in each NUT3 (Fig. 1). We then build a statistical model to relate PPP AS use to crop extents and climate variables deemed to have an explanatory power. Finally, we use the model to extrapolate PPP AS use from the regions of these countries to all other EU countries for which information is not available.

Data on the use or sales of individual PPP AS were collected for a number of European countries at the finest spatial scale feasible, as detailed in12; the gathered data are listed in Table 1 and Table SI1. Available national data on the use or sales of individual active substances were collected for 8 Member States (Belgium, Denmark, France, Germany, Ireland, Italy, The Netherlands, and Spain). In addition, available data for the United Kingdom (UK) were used for validation purposes as they were received after the modelling and cross-validation had been completed.

**Table 1:** Data availability for each country with source, spatial resolution and temporal coverage.

Country	Spatial Resolution	Temporal Coverage	URL
Belgium	Country level	2011–2017	https://fytoweb.be/fr/plan-de-reduction/vigilance/donnees-de-vente
Denmark	Country level	2012	http://www2.mst.dk/Udgiv/publikationer/2014/12/978-87-93283-33-6.pdf
France^3^	Province level	2010–2017	http://www.data.eaufrance.fr/jdd/bd45f801-45f7-4f8c-b128-a1af3ea2aa3e
Germany	Province level	2017	N/A
Ireland^4^	Country level	2011–2015	https://www.pcs.agriculture.gov.ie/sud/pesticidestatistics/
Italy^5^	Region level	2012	https://www.sian.it/
The Netherlands^6^	Country level	2012 & 2016	https://opendata.cbs.nl/statline/#/CBS/nl/dataset/84010NED/table?dl=1CE23
Spain^7^	Province level	2013	https://www.mapa.gob.es/es/estadistica/temas/estadisticas-agrarias/agricultura/estadisticas-medios-produccion/fitosanitarios.aspx

Although the amounts of AS PPP sold on the market and the amounts used in the fields may not exactly match, we assume that they are equivalent. Information on the authorized use of pesticides on specific crops or group of crops was obtained from pesticide labels where available. In some cases, we were able to rely on technical documents provided by Member State experts, as reported in^12^.

Once the information on PPP AS use was available for each NUTS3 region, we analysed its statistical relationships with crop extents and climatic variables. Given that the reported data refer to different years between 2011 and 2017 (Table 1), we made the practical working assumption that all data are representative of current pesticide use, representative of the situation around 2015. Therefore, our estimates cannot be linked to a specific year and are only valid under the assumption that the variation in pesticide use from year to year is not significant compared to the variability among crops, countries and NUTS3. For countries providing a time series of use data, we considered the average of the available years.

Information on agricultural land cover was extracted from the Corine Land Cover (CLC) dataset, which is produced as part of the Copernicus Monitoring Service and refers to the land cover/land use status of the year 2018 (CLC2018)^15^ at a spatial resolution of 100 m. (https://land.copernicus.eu/pan-european/corine-land-cover/clc2018).

A first stage of data processing was harmonization. Where data were provided as commercial pesticide products, the data were converted to PPP AS by computing the effective kilograms according to the percentage content of the active ingredient(s) in the pesticide products. Where AS sales were provided for more than one year, the average value was calculated.

In some countries, the data were already provided at the spatial resolution of NUTS3 level regions; where data were available at a coarser spatial resolution, they were disaggregated to NUTS3 level regions as described in^12^.

The spatialized emissions for each pesticide were also disaggregated within six different classes of land cover: arable land, fruit tree plantations, vineyards, olive groves, rice fields, and grassland.

We assigned a particular pesticide to a land cover class depending on whether the crops it was applied to were compatible with that land cover. In the supplementary material, table SI2, shows which EUROSTAT crop categories are associated with each of the six land cover classes. We took into account that some PPP AS were used almost exclusively in one of these main land cover classes; if they were used in more than one land cover class, they were distributed among them based on the manufacturer’s references (using the recommended quantities for each agricultural family) as explained in^12^.

The resulting quantities of 309 PPP AS at 770 NUTS3 for the 6 specific Corine Land Cover classes were stored into a dataset (represented in red Fig. 1).

In order to estimate the quantity of PPP AS applied in each of the 6 land cover classes within each NUTS3 region in the EU, we build statistical models using two types of predictors: crop extents (represented in green in Fig. 1) and climatic variables (represented in blue). Regarding crop extent, emissions were not consistently reported across all crops and the entire area. As an illustration, certain countries may report the amount of one AS for wheat, but not for barley. Consequently, we utilized two distinct crop extent datasets:

- Crop extents where the PPP usage was reported by the eight countries; this dataset was employed for the calibration of the prediction models.
- Total crop extents, regardless of whether the amount of AS applied was known or not, which were used for the prediction.

In both cases, crop extents were referred to the classes of crops in EUROSTAT (44 crop codes listed in the supplementary material Table SI2). The correspondence of these codes with crop names can be found in EUROSTAT (Annual crop statistics 2023 Edition. https://ec.europa.eu/eurostat/cache/metadata/Annexes/apro_cp_esms_an1.pdf)

Climate data were extracted from the PERSAM datasets (EFSA Spatial Data Version 1.1: https://esdac.jrc.ec.europa.eu/content/european-food-safety-authority-efsa-data-persam-software-tool) by the Joint Research Centre (JRC) for the European Food Safety Authority (EFSA)^16,17^. These datasets include grids of monthly mean temperature, monthly mean precipitation (mm/month), annual mean temperature, annual mean precipitation and the Arrhenius weighted annual mean temperature for the period 1950–2000 at a spatial resolution of 1 km. More details on these data can be found in^18,19^. For each of these variables, summary statistics of minimum, maximum, median, mean and standard deviation were calculated for each NUTS level 3 spatial unit. The above steps resulted 120 climatic variables and between 1 (rice fields or olives groves agricultural class) and 32 (arable agricultural class) variables related to the area devoted to each crop (supplementary material, table SI2) to be used as predictors of use of each PPP AS, for each land cover class.

Using the 120 climatic variables as predictors into the model makes it prone, among other problems, to extreme collinearity^20^. It would also lead us to overfitting, as the regression models would have too many explanatory variables for the number of observations available (at most 770, one in each NUT3 region). A selection of explanatory variables (feature selection) was made to avoid these problems^21,22^.

As for the explanatory variables related to the area devoted to each crop, several crops were grouped into a single explanatory variable of the area devoted to the whole set (e.g. cereals), under the assumption that the doses of an AS applied were similar for all the crops in the group [see^12^]. This means that the models considered the same applied amount of an AS for all crops in the group, and also excluded crops with low spatial coverage to which no ASs are applied. From the 44 initial crop categories (Table SI2), we selected the crops of Table 2 for consideration in the prediction models. It can be seen that the land cover class where this selection resulted in the largest reduction of crop classes is arable land. In this class, the area of all crops not considered is less than 1% of the total. Overall, we reduced the number of crop extent variables from 44 to 16.

**Table 2:** Main Arable CORINE Crop Classes considered for the modelling phase.

Agricultural Class	Crop Class	Id
Arable land	Cereal grain	C0000
Arable land	Plants harvested green	G0000
Arable land	Industrial crops	I0000
Arable land	Dry pulses and protein crops	P0000
Arable land	Fallow land	Q0000
Arable land	Root crops	R0000
Arable land	Fresh vegetables and strawberries	V_S0000
Arable land	Seeds and seedlings	E0000
Arable land	Nurseries	L0000
Arable land	Ornamental plants and Kitchen gardens	OTHER_ARA
Fruit tree	Fruits, berries and nuts	F0000
Fruit tree	Citrus fruits	T0000
Grassland	Permanent grassland	J0000
Olive groves	Olives	O1000
Vineyards	Grapes	W1000
Rice fields	Rice	C2000

For the climatic variables, we apply a pairwise correlation pre-evaluation^23^. This method computes pairwise correlations between all variables and identifies pairs of variables with a correlation coefficient above a certain threshold. For each highly correlated pair, the method computes a score that reflects the contribution of each variable to the overall correlation. The variable with the lower score is then considered less important for the analysis and is removed. This process is repeated until no highly correlated pairs remain, resulting in a smaller set of variables that are less likely to be collinear. This process reduced the set of climate variables from 120 to 12. Therefore, we used a total of 28 variables as candidate predictors of PPP AS use.

In the modelling phase, a second stage of feature selection is carried out for each AS, based on the multiple regression model in which area and climatic variables are considered simultaneously. A StepAIC procedure^24^, based on Akaike’s Information Criterion^25,26^, balances the goodness of the model fit with the number of parameters required to achieve that fit.

### Pesticide prediction modelling/estimation for European Union regions

The multiple linear regression paradigm was applied as the prediction method because, as a predictive tool, it allows us to explain the relationship between many independent variables (X_1_, X_2_, X_p_) and the dependent variable (Y) being tested^27^. The learned relationships are linear and can be written for a single instance as follows:

$${\rm{y}}={{\rm{\beta }}}_{0}+{{\rm{\beta }}}_{1}{{\rm{x}}}_{1}+{\rm{\ldots }}+{{\rm{\beta }}}_{{\rm{p}}}{{\rm{x}}}_{{\rm{p}}}+\in $$

Linear models predict outcomes based on the weighted sum of the features, with the weights represented by β_j_. The first weight (β_0_) is the intercept and is not multiplied by a feature. These models are computationally efficient and easy to interpret because increasing *x*_*k*_ by one unit increases the prediction for y by β_k_ units. This is important because the process is repeated multiple times (308 PPPs, 6 agricultural classes (Table 2), several configurations of linear models).

**Fig. 2:**
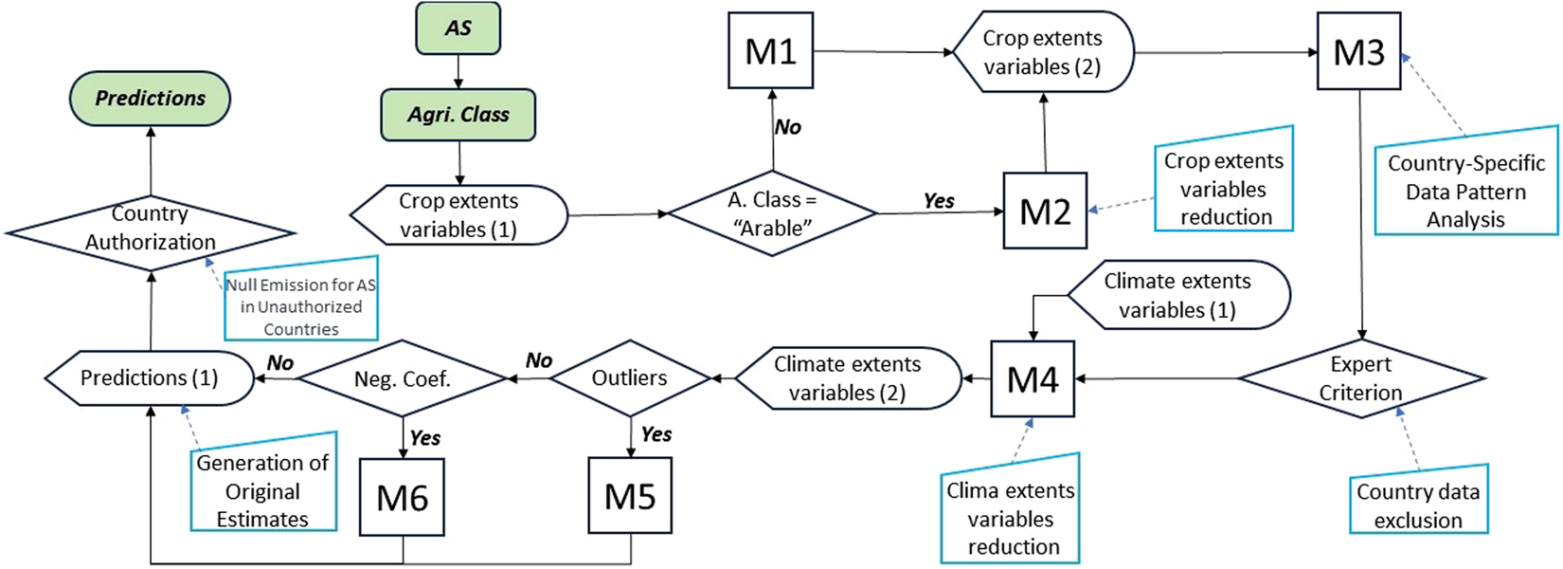
Flow chart illustrating the use of the different models according to the context of each Active Substance (AS) and agricultural class, as well as the generation of the final prediction.

The statistical programming language R^28^ was used to implement an automated process designed to apply a sequence of regression models to each dataset. A data set is the data relating to a PPP AS for one of the main LCC (arable land, fruit tree plantations, vineyards, olive groves, rice fields, grassland). The automated process produces the following sequence of models as described in the flow chart of Fig. 2:

- M1: A multiple regression model that considers the crop extents where an AS is applied as explanatory variables.
- M2: A multiple regression model with the selection of the StepAIC explanatory variables from the previous set.
- M3: A multiple regression model, in which an additional explanatory variable^29^, i.e. the country to which each data belongs, is added to the M2 explanatory variable set.
- M4: A multiple regression model including the climate-related explanatory variables in addition to crop extents.
- M5: A model using robust estimation regression methods^24^, to reduce the effect of outliers.
- M6: A regression model with restrictions^30^, in which negative coefficients are not allowed for the explanatory variables and which aims to minimize the value of the intercept (which would imply applying AS when the agricultural area is zero) (M6).

For the quantitative assessment of all the models created in this study, cross-validation was used to evaluate their performance and detect overfitting^31^. Cross-validation is a resampling procedure that divides the data sample into k groups and selects one group as validation data and the remaining k-1 groups as training data. The process is repeated for each group, resulting in k times of cross-validation. In this study, a 10-fold cross-validation technique was used, which was repeated 100 times. In addition, a country-specific cross validation was performed to help numerically detect whether the data from one country showed very different patterns from the rest. Relative or normalized metrics were used for comparing different PPP ASs (with markedly different reference values).

**Fig. 3:**
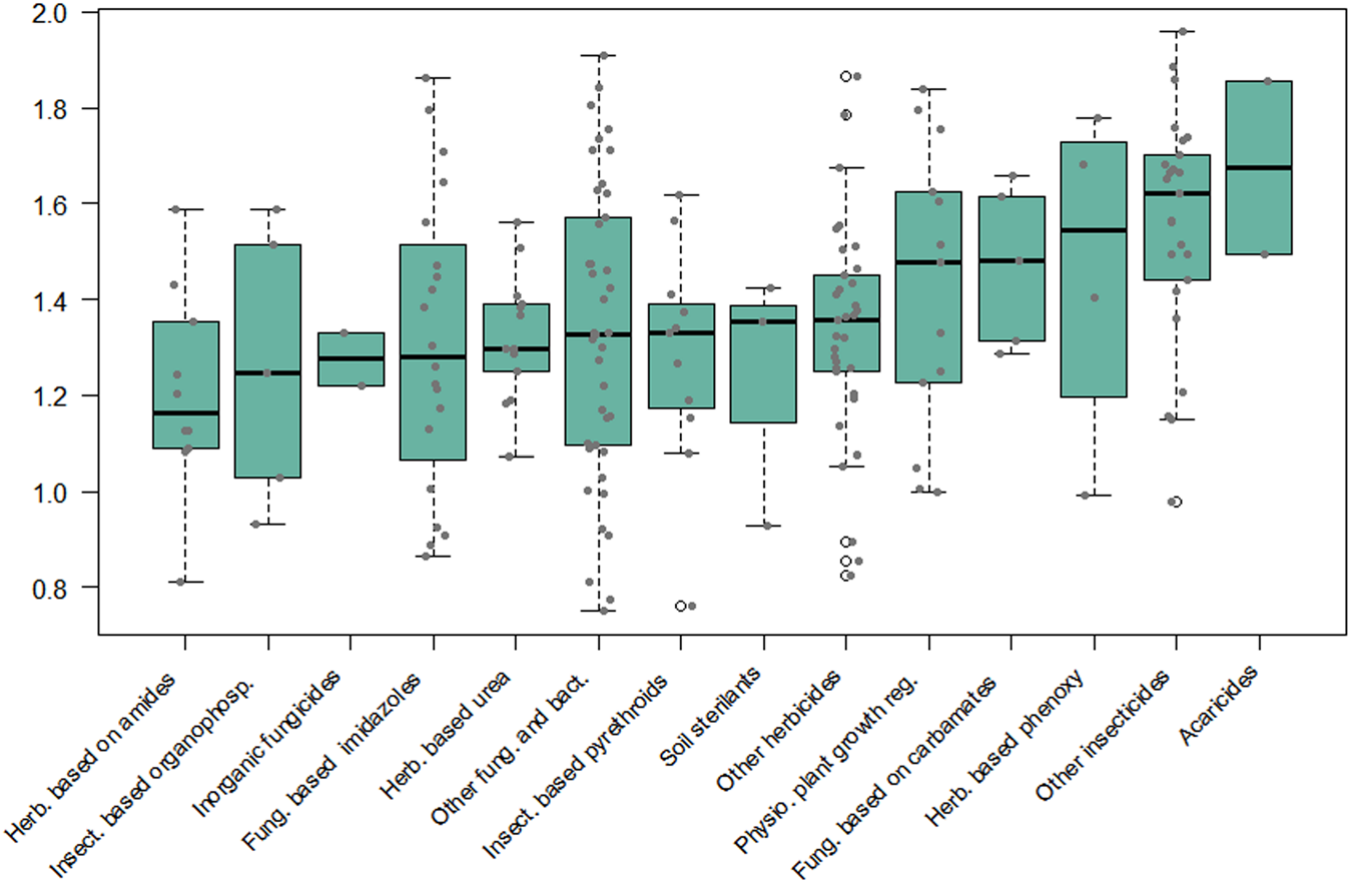
Boxplots by AS categories presenting the statistical distribution of model prediction quality based on the Symmetric Mean Absolute Percentage Error (SMAPE) metric. The data reflect the results of the 10-fold cross-validation process for the best model in each AS applied to the arable class. For the SMAPE metric, a result closer to zero indicates a better outcome.

**Fig. 4:**
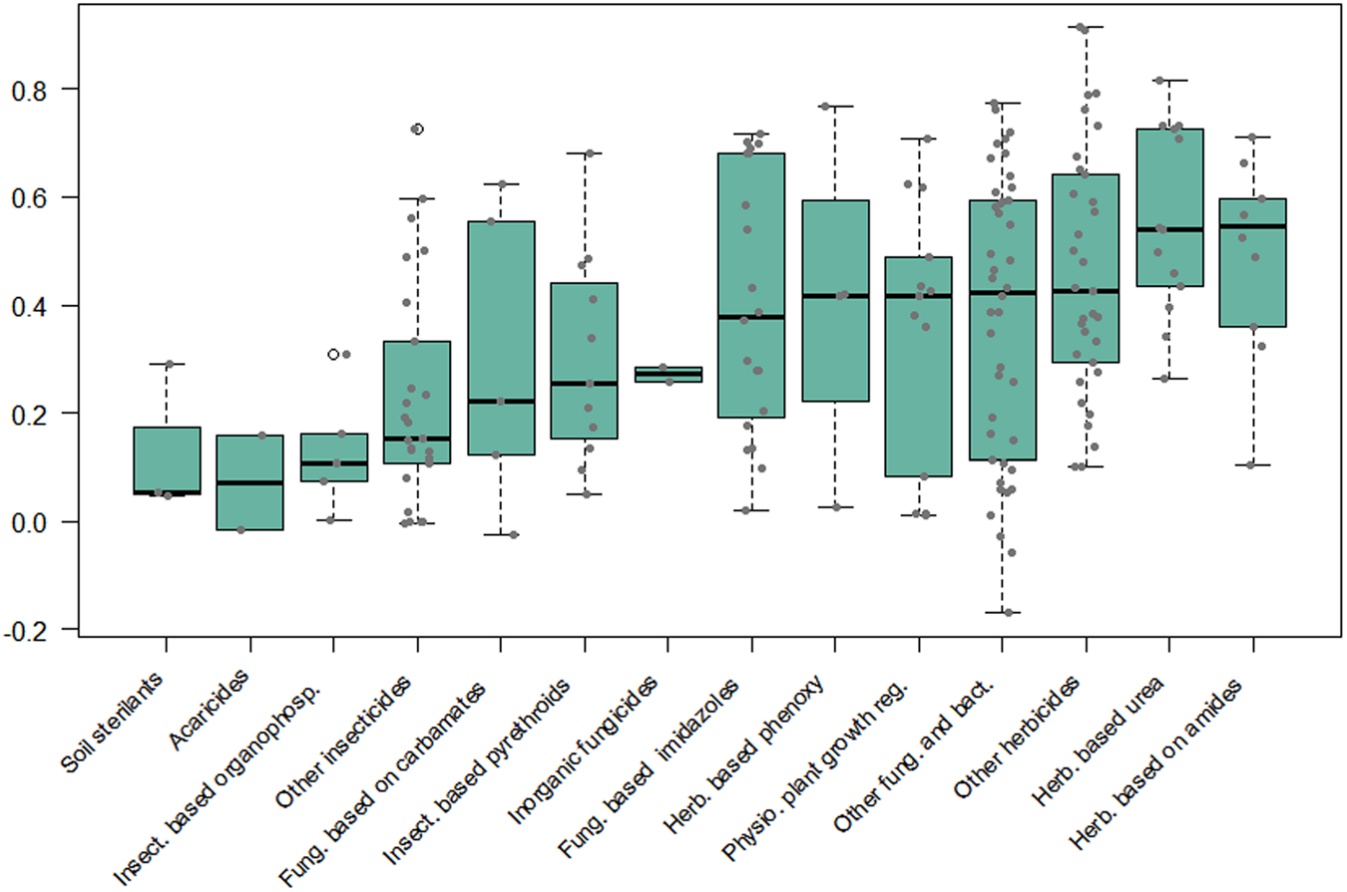
Boxplots by AS categories presenting the statistical distribution of model prediction quality based on the NSE (Nash–Sutcliffe model efficiency coefficient) metric. The data reflect the results of the 10-fold cross-validation process for the best model in each AS, applied to the arable class. For the NSE metric, a result closer to one indicates a better outcome.

There is no single best metric to quantitatively compare model output to observational data and evaluate the performance of a regression model^32^. Different metrics compare the performance of models in absolute (more suitable for different models for the same AS) or relative term (more objective for predictions for different AS). In the cross validation process we employed several metrics, namely Symmetric mean absolute error (SMAPE, Fig. SI3), Nash-Sutcliffe Efficiency (NSE, Fig. SI5), normalized root mean squared error (NRMSE, Fig. SI2), determination coefficient (R2, Fig. SI1), and normalized mean absolute error (NMAE, Fig. SI4) to assess model goodness-of-fit. For example, Figs. 3, 4 show respectively the SMAPE and NSE values calculated by cross-validation of the AS predictions for the “arable land” class. Depending on the quantity and nature of the data as well as of the models, values between 0.0 and 1.0 for NSE (Fig. 4) indicate that the model performs better than using the simple mean^32^. Despite the high variability in performance among the ASs of the same group, the predictions of *herbicides based on amides* seem to be among the best, while predictions for *acaricides* and *other Insecticides* seem to be among the worst (Figs. 3, 4).

In practical terms, the M2 model was only used to reduce the number of variables relating to crop- specific areas in the “arable land” agricultural class (Table 2) since in all other agricultural classes we have only one or two crops as predictors, hence the M2 model would be identical to M1. The sequence of models (see Fig. 2) continues for all agricultural classes, from the M2 or M1 output depending on whether it is arable land or another agricultural class. To analyze whether PPP AS use displays significant variations among countries where data was collected, the M3 regression model including the country as an explanatory variable was utilized. In this case, extrapolation to other countries might be limited, as use patterns would be country-specific. The M3 model is never used for the extrapolation of PPP AS use to other countries. The M4 model was mainly applied to check the improvements obtained by including climatic variables and selecting the most suitable ones (generally less than 4). For agricultural land classes except “arable land”, the M4 model predictions were often accepted and the model sequence was closed.

When the PPP AS was not widely used in the 8 reference countries, the decision to accept or reject the models becomes more challenging. We limited the analysis to those PPP AS that were used at least in 4 out of 8 reference countries.

**Fig. 5:**
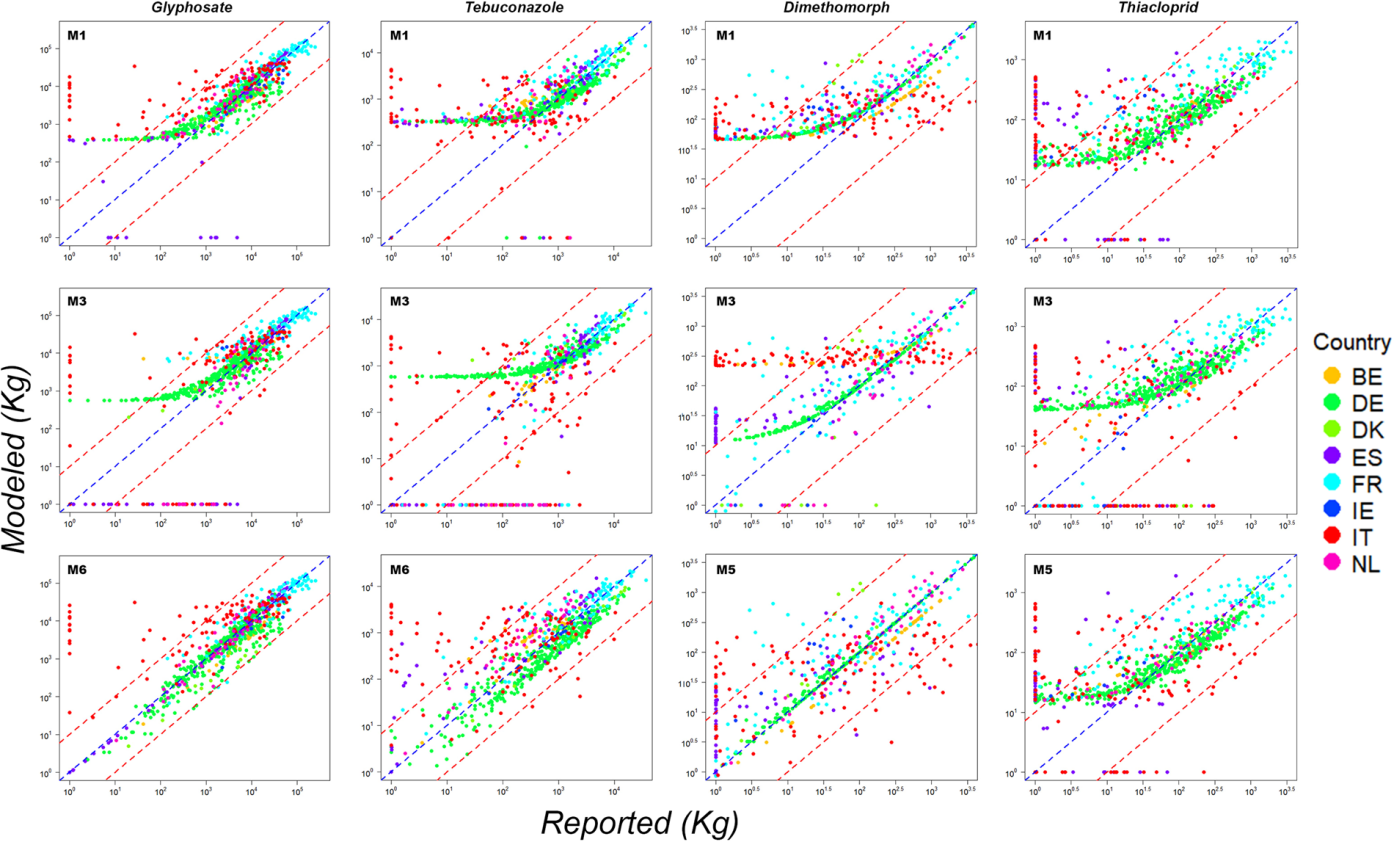
Comparison of reported versus modelled values for four AS: Glyphosate, Tebuconazole, Dimethomorph and Thiacloprid (arranged in columns from left to right). The predictive models used for comparison are denoted as follows: M1 in the first row, M3 in the second row, and either the M5 model (for Dimethomorph and Thriacloprid) or the M6 model (for Glyphosate and Tebuconazole) in the third row. To display in logarithmic scale, all values (both reported and modelled) have been incremented by 1 kg. The red dashed lines indicate the 1:10 and 10:1 ratios. Data for each country are shown in a different colour.

For the agricultural class “arable land”, which encompasses over 65% of the total applied AS by weight, the initial set of explanatory variables was large, and included the extent of land of 10 different crops (Table 2). The initial model complexity was thus higher than in the other agricultural class. In these cases, predictions were sequentially examined for all models, from M1 to M6. Differences in predictions between the M4 and M5 models were examined to identify and rectify outliers in NUTS3 regions, which had a considerable impact on the predictions. For the most commonly used active substances (those accounting individually for more than 1% by weight within their respective categories) the occurrence of outliers was frequent. Therefore, the final prediction was carried out using the M5 model. Finally, the coefficients of the explanatory variables in M5 models were further examined. This analysis served two purposes: to gauge the effect of atypical observations on each explanatory variable, and to detect instances of negative values, which lacked physical justification. In such circumstances, the predictions were based on model M6. Let us consider two specific examples: *Dimethomorph* and *Thiacloprid*. In the M4 model, most of the coefficients for the explanatory variables show a positive coefficient, but certain data points stand out as anomalies. In this scenario, the predictive performance of the M5 model appears more robust than that of the M6 model. This observation is supported by insights gained from Fig. 5, along with the corresponding data provided in Table 3.

**Table 3:** Goodness of fit metrics, namely NRMSE (Normalized Root Mean Square Error) and Nash-Sutcliffe Efficiency (NSE), for models M1 through M6 with respect to five active substances (AS): Glyphosate, Tebuconazole, Dimethomorph, Thiacloprid, and Esfenvalerate.

	Glyphosate		Tebuconazole		Dimethomorph		Thiacloprid		Esfenvalerate	
	NRMSE	NSE	NRMSE	NSE	NRMSE	NSE	NRMSE	NSE	NRMSE	NSE
M1	0.463	0.786	0.536	0.712	0.653	0.572	0.616	0.620	0.827	0.316
M2	0.463	0.785	0.536	0.712	0.654	0.571	0.617	0.619	0.828	0.314
M3	0.451	0.797	0.518	0.732	0.622	0.613	0.602	0.637	0.677	0.541
M4	0.440	0.806	0.500	0.750	0.603	0.636	0.589	0.653	0.780	0.391
M5	0.504	0.745	0.610	0.628	0.671	0.549	0.656	0.570	0.985	0.028
M6	0.472	0.777	0.579	0.664	0.654	0.571	0.660	0.564	0.840	0.294

Although the process of generating all models for each AS and land cover class, as well as verifying assumptions of normality and homogeneity of residual variance (homoscedasticity) and identifying possible outliers, was automated, ultimately the decision of which model to select remained somewhat subjective (expert judgement), based on both graphic and quantitative evaluations. In such cases, modifications to linear models were applied (variable transformations, weighted least squares, outlier removal, etc.).

Whichever model was selected as best to make the final estimate of an AS, we checked the authorisation to use each AS in each country (as per the EU Pesticides database: https://ec.europa.eu/food/plant/pesticides/eu-pesticides-database/active-substances/?event=search.as), and assigned null emission when an AS was not authorised.

Figure 5 presents illustrative graphs juxtaposing observed and modeled values for four active substances (AS), namely *Glyphosate*, *Tebuconazole*, *Dimethomorph*, and *Thiacloprid*. The contrasts among the outcomes generated by the respective models M1 (top row of Fig. 5), M3 (middle row in Fig. 5), and the ultimate estimation model (M6 or M5, bottom row in Fig. 5) adopted for these AS are conspicuously evident for each of these substances. These visual representations offer rich insights; for instance, they reveal that the performance in the M3 model for *Dimethomorph* is particularly variable across countries, or highlight the selected final model’s tendency to overestimate the application quantities of *Glyphosate* in Italy, as well as the notable variability in the performance of model predictions for *Thiacloprid*, which indicate substantial application amounts in Spain and Italy where null applications are reported, while conversely predicting null applications in areas where substantial quantities are reported.

*Glyphosate* is the most widely used herbicide, comprising 37% of total herbicides by weight. Based on both graphical analysis (Fig. 5) and statistical performance metrics (Table 3), the M6 model was determined to be the best predictive choice specifically for *Glyphosate*. The next 30 most commonly employed herbicides AS across the eight reference countries collectively contribute to 93% of herbicide use by weight. Over 95% of these herbicides, in terms of weight, are primarily applied to the “arable land” class. *Glyphosate*, however, is utilized solely in 62% of “arable land” class. This makes the overall predictions of these herbicides predominantly reliant on the estimation of the “arable land” agricultural class. Predictive models were constructed for these 30 active substances, resulting in metrics closely resembling those of *Glyphosate*, albeit with exceptions such as *Terbuthylazine* and *Dimethachlor*, where predictions demonstrated comparatively lower quality.

In general, predictions were primarily carried out through the utilization of models M5 or M6. Among the group of the 30 least-utilized herbicides (together accounting for a mere 0.4% of total herbicide use), models proved unattainable for 25 due to their exclusive deployment in only one or two of the eight countries. For the remaining herbicide substances, accounting for approximately 6.5% of the total applied quantity, model performances deemed inapplicable.

A similar outcome is found when modelling fungicides, albeit with a higher frequency of exceptions. The 30 most demanded active substances in the category collectively account for 90% of the demand in the eight reference countries. In this context, application within the “arable” class is less common than for herbicides: 6 of these 30 most consumed active substances, use in the “arable land” class remains below 60% of the overall use. Models designed for the “arable land” class showed sub-optimal quality for 4 of them (i.e. *Captan*, *Thiram*, *Metiram* and *Dithianon*) compared to the remaining 30 most consumed fungicides. In contrast, within the 30 least consumed fungicides (which together contribute to 0.8% of total use), models could not be generated for 18 because they were only used in one or two of the eight reference countries.

Two examples from the fungicide category, namely *Tebuconazole* (constituting 4.5% by weight of the category, with 93% application within the ‘arable’ land category) and *Dimethomorph* (comprising 0.9% by weight of the category, employed at a rate of 51% within the ‘arable’ class), have been included in Fig. 5 and detailed in Table 3. For *Tebuconazole* and *Dimethomorph*, the ultimate predictions were derived through the utilization of the M6 and M5 models respectively (see Fig. 5, Table 3 and Table SI3).

In other categories of PPP (such as insecticides, plant growth regulators or “soil sterilants”, “acaricides”) characterized by a smaller number of active substances (AS) and lesser use, the situation mirrored that observed in herbicides and fungicides. Approximately 30% of the AS in each category accounted for roughly 90% of the total use, whereas another 30% collectively accounted for less than 1%. Models could not be formulated for the majority of AS within the least-demanded 30%, primarily due to their exclusive application in just one or two countries among the reference set.

For the most part, models for the “arable land” class associated with the top 30% of AS were considered acceptable, often in the framework of the M5 or M6 models. An example of an AS in this category is *Thiacloprid* (which constitutes 12% of the total demand within the Insecticides category), for which results for some models can be examined in Fig. 5 and Table 3.

Crop areas are always the most important explanatory variables for AS use. As the agricultural class “arable land” corresponds to 10 crops, it poses the highest level of uncertainty in predicting pesticide usage in this class. In contrast, the “fruit trees” class corresponds to two crops and the remaining classes to a single crop (Table 2). Prediction models for pesticide use on olive groves, rice fields, grassland, fruit trees and vineyards usually present less uncertainty in the prediction for most AS, as they only need to consider one variable related to crop.

Estimates of AS that are widely used in all eight reference countries are generally more reliable than those for which application data are either unavailable or completely absent in some of the reference countries. The modelling process of generating and estimating was performed on 308 ASs, for which partial information regarding their application had initially been successfully collected. In particular, quite a number of ASs were excluded from the predictions in the public dataset, so our predictions were considered meaningful only for 152 ASs. The vast majority were excluded because the number of reference countries to build the model was very low and the uncertainties in predictions were high.

In addition, it is important to note that the predictions for Northern and Eastern European countries have a higher degree of uncertainty, due to the absence of usage data in these regions: where AS usage patterns and regulations may vary significantly compared to Southern and Western Europe. Cautious interpretation of the estimates is recommended, recognizing the constraints imposed by data availability and the need to take into account unverified assumptions in divergent geographical contexts.

## Data Records

A dataset^33^ stored in.csv format for easy use in any statistical software. The dataset contains estimated emissions of 152 active substances at NUTS Level 3 European administrative units^34^. Each dataset contains estimates for active substances belonging to one of the main groups: Fungicides and Bactericides, Herbicides, Insecticides and Acaricides, Plant growth regulators, Molluscicides, Other plant protection products. The estimation was carried out independently for each active substance on the basis of data on sales of active substances provided by 8 Member States (Table 1). The spatial resolution and extent of the analysis corresponded to the NUTS3 regional level for Europe.

The dataset includes the predicted use of the active substance (kilograms) per region, the common name, the CAS (Chemical Abstracts Service Council number identifier), the CIPAC (Collaborative International Pesticides Analytical Council number identifier), the ID_EUPDB (identifier in the European Union Database of pesticides) and the Chemical Class for each active substance.

The dataset contains the following variables:

- *Country*: country ISO-3 code.
- *NUTS3*: Level 3 European administrative units.
- *Categories of products*: category to which the active substance belongs.
- *Chemical Class Substance*: active substance chemical class.
- *ID_EUPDB*: active substance identifier in the European Union Database of pesticides.
- *CAS*: Chemical Abstracts Service Council number identifier of the active substance.
- *CIPAC*: Collaborative International Pesticides Analytical Council number identifier of the active substance.
- *Substances common names*: active substance common name.
- *KG_TOT*: estimated total emission in kg.

## Technical Validation

### Data completeness assessment

The quality of the Europe-wide emission prediction for each of the PPP AS included in the dataset depends, among other things, on the number, distribution and reliability of the reference values on which the models are built. The information from the 8 reference countries was not always complete. In many cases the quantities applied for some crops or for certain areas were not provided. A first check of the completeness of the data comes from a comparison with EUROSTAT data. These data cover sales volumes (in kilograms per annum) of pesticides aggregated by broad groups. Table 4 summarizes, for each of these groups, the number of AS within the group and the level of completeness of the available information.

**Table 4:** Some indicator related with data completeness by pesticide group.

Pesticide groups	PPP AS Reported by EUROSTAT (1)	PPP AS for 8 Countries (2)	Total kg PPP AS in 30 EU Countries (3)	Total kg PPP AS in 8 Countries (4)	Reported kg PPP AS in 8 Countries (5)	% Sales Volume in 8 Countries (6)	Application Area (Mha) in 8 Countries (7)
Inorganic Fungicides	7	5	87.6	76.6	4.90	6.4	14.96
Other fungicides and bactericides	47	34	38.6	23.3	14.7	63.0	56.80
Fungicides based on carbamates	9	6	25.1	17.9	12.3	68.7	34.1
Fung. based imidazoles and triazoles	24	13	10.3	5.8	4.80	82.3	44.6
Fungicides based on benzimidazoles	2	1	1.93	0.7	0.38	54.6	26.3
Fungicides based on morpholines	3	2	1.59	1.18	0.81	68.9	23.8
Other herbicides	47	35	78.79	49.01	29.1	59.4	66.17
Herb. based on amides and anilides	15	9	19.64	12.1	9.62	79.4	40.41
Herb. based on derivatives of urea	22	8	8.74	5.35	2.39	44.7	43.45
Herb. based on phenoxy- phytohormones	6	5	8.67	4.2	4.07	96.8	61.69
Herb. based on triazines & triazoles	3	3	7.08	4.35	3.96	91.18	28.3
Herbicides based on dinitroaniline	3	2	4.96	3.36	2.00	59.5	36.14
Herbicides based on carbanates	4	4	1.65	1.08	0.78	72.99	9.58
Other Insecticides	28	26	25.61	22.03	1.02	4.65	50.43
Insect. based on organophosphates	7	7	5.11	1.91	2.3	120.86	54.55
Insecticides based on pyrethroids	13	11	1.48	0.79	0.47	60.2	56.33
Insecticides based carbamates	6	4	0.67	0.55	0.19	34.37	21.05
Soil sterilants (incl. Nematicides)	4	3	23.56	21.96	2.72	12.41	39.35
Physiological plant growth regulators	18	13	12.07	7.57	4.22	55.75	43.35

(1) Number of PPP AS reported by EUROSTAT

(2) Number of PPP AS in the group compiled for the 8 countries in this study

(3) Amounts in kg of all PPP AS in the group which, according to EUROSTAT data, are applied in 30 european countries.

(4) Amounts in kg of all PPP AS in the group which, according to EUROSTAT data, are applied in the 8 countries for which reference are available.

(5) Amounts in kg of all PPP AS in the group which is covered by the reported data of the 8 countries.

(6) Percentage of sale volumes according to EUROSTAT for the 8 countries for all PPP AS in the group that is covered by the reported data of the 8 countries.

(7) Area of application (Mha) of all PPP AS in the group which is covered by the reported data of the 8 countries

In this work, pesticide use data were collected for more than 60% of the PPP AS of each category for 16 of the 19 pesticide groups (see column labelled (1) in Table 4). The ratio between the AS amounts according to the data we have collected and the amounts applied according to the EUROSTAT was above 40% for 10, and below 20% for only in 3 of the 19 groups (column (6) in Table 4). Likewise, the ratio of the areas for which data could be obtained to the total agricultural area was above 40% in 10 of the NUTS3 regions and below 20% in only two of the regions (Table 4, column (4)). From the above comparison, the reported use of pesticides for the 8 countries considered tends to be smaller than the reported sales volumes of pesticides in the same countries although our data are within one order of magnitude of the EUROSTAT data.

### Comparison with the EUROSTAT country data

EUROSTAT data on annual pesticide sales, available at the national level and aggregated by major groups (insecticides, herbicides, fungicides, plant growth regulators, rodenticides, and others) and by product categories (a further disaggregation of the major groups) were used to validate our estimates. For this purpose, we aggregated all AS belonging to each product category and made comparisons by country.

Table 5 presents a comparative analysis of EUROSTAT reported values and model estimates across various pesticide categories and countries. The table highlights country-specific data for five categories: “Other Herbicides”, “Other Fungicides and Bactericides”, “Inorganic Fungicides”, “Herbicides Based on Amides and Anilides” and “Other Insecticides”. Each category is represented by two columns: the first column displays EUROSTAT reported values in kilograms (Kg), and the second column exhibits the modeled-to-reported ratio. This ratio, found in the ‘Mod’ column, signifies the relationship between model estimates in this study and the reported values. Notably, a ratio of 2 in the ‘Mod’ column suggests that the model predicts a quantity twice that of the EUROSTAT reported value. This comprehensive table offers insights into the comparison between modeled and reported pesticide quantities across different categories and countries.

**Table 5:** Comparison of EUROSTAT Reported Values and Model Estimates (Mod) by Country for Five PPP Categories.

Co	Other herbicides		Other fungicides and bactericides		Inorganic fungicides		Herbicides based on amides and anilides		Other insecticides	
	EUROSTAT	Mod	EUROSTAT	Mod	EUROSTAT	Mod	EUROSTAT	Mod	EUROSTAT	Mod
AT	607887	2.22	415514	2.15	1025249	0.03	240437	4.86	389024	0.10
BE	1348349	1.10	812590	0.75	289198	0.06	323314	1.95	460926	0.10
BG	1778175	1.34	576932	2.33	313068	1.12	283996	9.01	176249	0.64
CY	98386	0.45	23543	1.23	467942	0.20	1163	54.08	103078	0.02
CZ	1552536	1.47	656411	1.72	333004	0.06	645712	2.98	62761	1.31
DE	7825176	1.28	4433539	1.42	2682022	0.11	3584578	1.35	13425403	0.03
DK	2041936	0.31	220158	1.38	8319	0.00	79211	0.78	25424	1.28
EE	376302	1.67	41313	7.14	0	—	42137	2.12	8916	0.89
EL	1026563	2.15	312863	4.19	843496	2.92	118475	17.19	571984	0.17
ES	12141199	0.51	2459844	1.60	32732351	0.09	814780	3.71	5013083	0.05
FI	804508	2.28	3292227	0.23	0	—	6595	7.55	0	—
FR	17392470	0.73	8666411	0.68	14536112	0.00	5946047	0.72	2375237	0.08
HR	390671	2.69	113811	6.04	515710	0.08	180758	4.45	80907	0.55
HU	2252575	1.35	837833	2.32	1913720	0.04	1032614	2.96	459828	0.32
IE	1429357	0.39	379743	0.41	6292	0.00	32003	10.41	5385	1.14
IT	5256013	1.48	4893292	0.69	26170063	0.15	1008100	1.54	619306	0.30
LT	771528	2.15	218988	4.46	0	—	253100	0.98	26979	1.83
LU	31817	2.27	22411	1.61	0	—	11794	3.60	0	—
LV	627111	1.51	94121	4.83	695	7.38	106874	1.59	27270	0.46
MT	3972	0.66	1545	5.96	94273	0.69	0	—	0	—
NL	1579917	1.18	1429833	0.45	138773	0.01	318264	0.88	102906	0.52
NO	424775	0.00	34019	0.00	17673	0.00	1573	0.00	4482	0.00
PL	6925858	1.38	3225089	1.96	427695	0.11	1538570	5.60	277209	1.61
PT	1552131	0.91	820500	1.04	2872629	0.42	145639	13.11	466156	0.14
RO	2564827	1.79	1408984	2.87	1043887	0.25	1145963	4.82	585148	0.56
SE	1666808	1.36	114949	4.30	3009	0.00	55309	2.01	28181	1.32
SI	124176	3.83	172256	1.79	478052	0.17	48191	4.33	35804	0.31
SK	620428	1.40	211289	2.99	155616	0.10	240018	4.19	31412	1.22
UK	5195639	1.84	2338918	1.32	60475	0.01	1352281	3.48	0	—

Reported values from EUROSTAT are denominated in kilograms (Kg), and the ‘Mod’ values represent the modeled-to-EUROSTAT ratio.

Depending on the product category being compared, the difference between EUROSTAT country sales data and our appropriately aggregated estimates, the results can be quite consistent, as for the product categories “other herbicides”, “other fungicides and bactericides”.

In the category “Other Herbicides”, constituting around 60% of the major group “Herbicides”, and “Other Fungicides and Bactericides “, accounting for roughly 24% of total fungicide usage by weight, our estimates often tend to surpass EUROSTAT sales data across most European countries (Table 5). On the other hand, certain categories like “Inorganic Fungicides” and “Herbicides Based on Amides and Anilides” exhibit larger discrepancies. For instance, “Inorganic Fungicides”, representing approximately 53% of total fungicide sales, shows significantly lower estimates than EUROSTAT data for the majority of countries (Table 5).

This mainly happens because this category includes copper compounds, which represent an important share of sale data, but which were not reported as being used in the 8 countries for which we retrieved the data and were therefore not modelled. For the category”herbicides based on amides and anilides”, which represents about 15% by weight of the “*herbicides”* group, there is a tendency to overestimate compared to the EUROSTAT statistics (Table 5).

For the product category”Other insecticides” which represents 78% of the major group “Insecticides”, there are minor differences compared to EUROSTAT statistics in several countries (BG, EE, RO, NL, PL, CZ, etc.), while in other countries (FR, ES, DE) the underestimation exceeds 2 orders of magnitude (Table 5). This is striking, as these countries are part of the group of 8 countries that have provided the data to build the European prediction models.

In general, the differences between our estimates of PPP AS use and EUROSTAT sales data are acceptable, considering that:

- The differences in reported pesticide use among the 8 countries for which data were available to make the prediction were already significant.
- There are other factors that we have not included in the model, such as the type of agricultural practices, soil type, and behaviour of farmers, which will certainly also have an influence on the application of PPP AS, both in terms of total amounts and the distribution within the different AS in each category.
- Some PPP AS are used in a very specific way in only 1 or 2 crops, with patterns that may vary from one country to another.

Considering all these limitations, it is unlikely that the use of individual PPP AS can be estimated more accurately at the EU level in the absence of more detailed data^35^.

### Comparison with independent data for the United Kingdom

For an independent assessment of our estimates, we used PPP AS use data for the UK, which can be accessed online (https://pusstats.fera.co.uk/home). The UK information consists of data disaggregated for 12 NUTS Level 1 regions for 253 PPP AS. Of these 253 AS, 124 are within the 152 for which we have been able to make the highest quality predictions, and which are included in this dataset. Since our predictions were made at a smaller spatial resolution (NUTS Level 3), our predictions were aggregated to NUTS Level 1 spatial resolution for this comparison.

Three visual representations have been generated to provide a concise overview of the comparison between the four categories of pesticides, namely herbicides, fungicides, insecticides, and plant growth regulators. These categories were selected based on the availability of UK emissions data for a significant number of active substances.

**Fig. 6:**
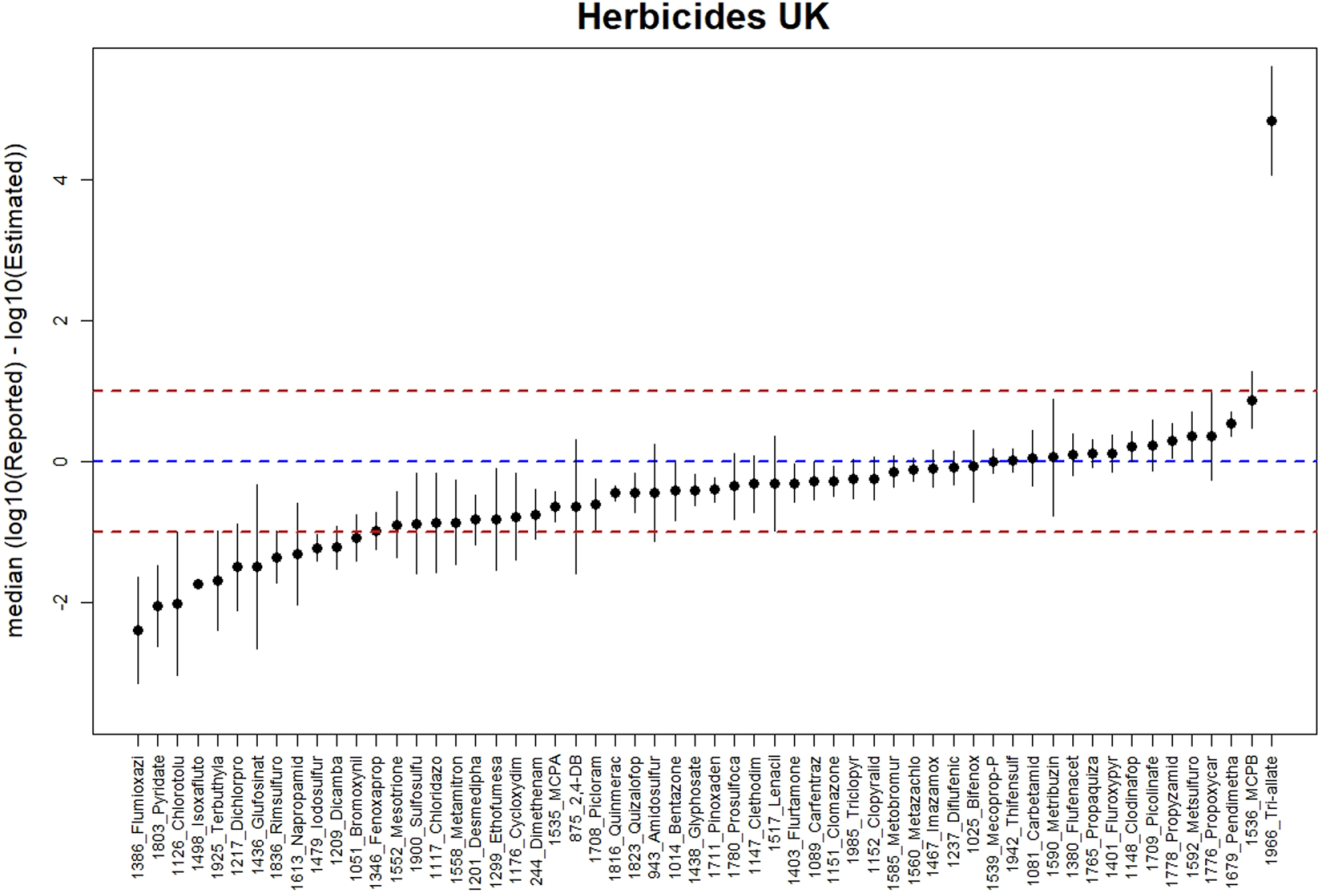
Vertical lines show the comparison between the estimates and the reported UK emissions for 59 AS of the Herbicide category in 11 UK regions. The dot in each vertical line is the median of the difference between log10 reported values and log10. The vertical lines show the median absolute deviation, as a measure of the robust error in each estimate. The blue dashed horizontal line indicates 1:1 relation. The red dashed horizontal lines indicate the 1:10 and 10:1 relationship.

For herbicides it can be seen (Fig. 6) that for most of the ASs in the category (48 out of 59) the deviations of our estimates from the reported values are less than one order of magnitude, with somewhat more of a tendency to overestimate.

*Triallate* shows a significant underestimation (Fig. 6). This AS is not authorised in most EU countries, hence very low applied amounts are reported in the 8 reference countries, while it is still in use in the UK. On the other side, *Flumioxazine* shows a significant overestimation (Fig. 6 left). This deviation is probably due to the fact that *Flumioxazine* is one of the least used herbicides (approximately 0.06% of the total by weight), its model was generated with application data from only 3 of the 8 reference countries and for which only 2 of the 11 UK regions report application rates.

**Fig. 7:**
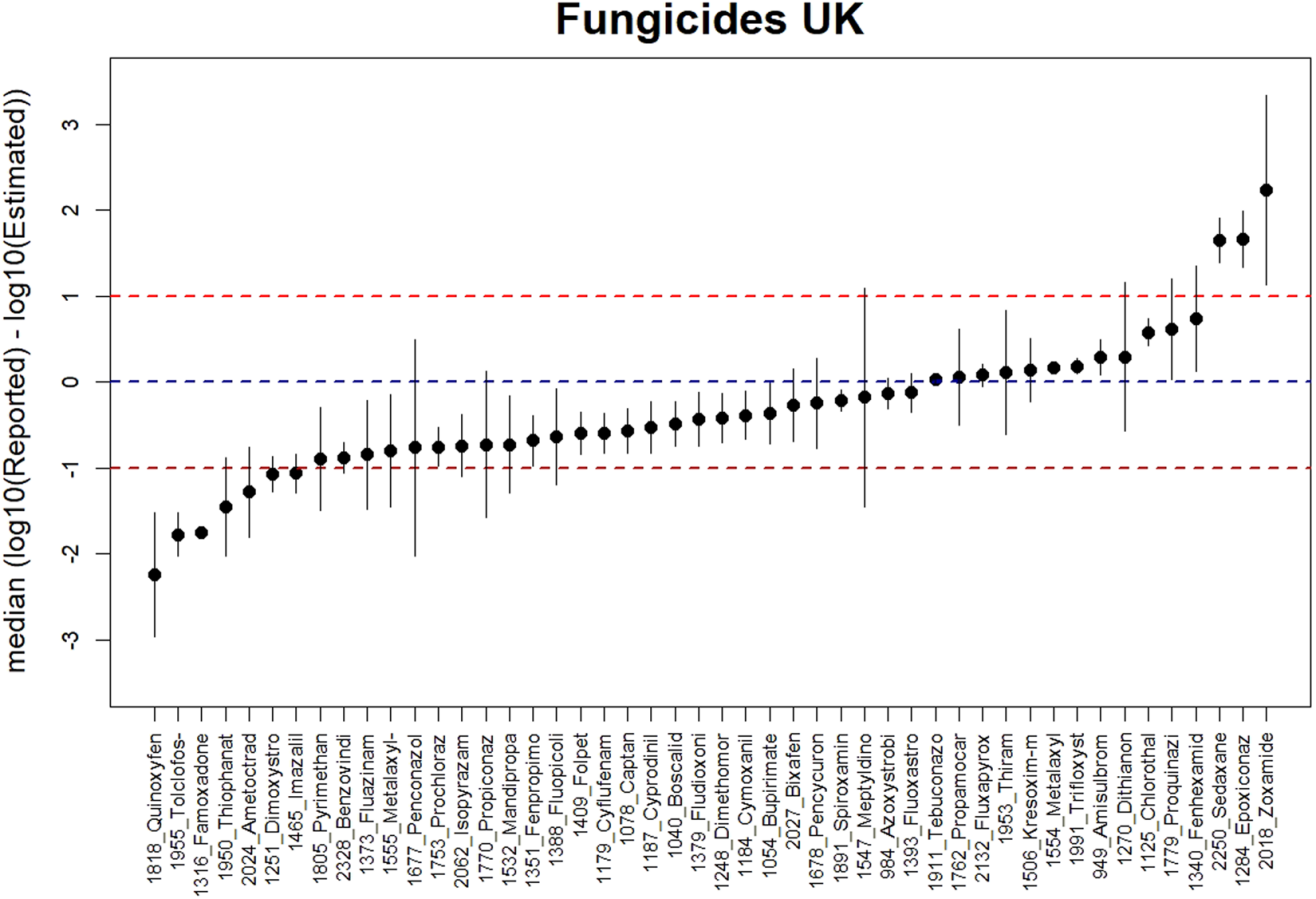
Vertical lines showing the comparison between the estimates and the reported UK emissions for 49 AS of the Fungicides category in 11 UK regions. The dot in each vertical line is the median of the difference between log10 reported values and log10. The vertical lines show the median absolute deviation, as measure of robust error in each estimation. The dashed blue horizontal line indicates 1:1 relation. The red dashed horizontal lines indicate the 1:10 and 10:1 relationship.

Similarly, for fungicides (Fig. 7) a large part of the estimates (41 out of 49) are within one order of magnitude of the values reported in the UK, with a greater tendency to overestimate.

*Zoxamide* is the fungicide with the largest underestimation (Fig. 7 right), but in this case this is not due to the fact that AS is not authorised for application in the United Kingdom. Likewise, the largest overestimation is for *Quinoxyfen* (Fig. 7 left), which, although only accounting for approximately 0.1% of the total fungicide by weight, is available in reference values for use in 9 of the 11 UK regions.

**Fig. 8:**
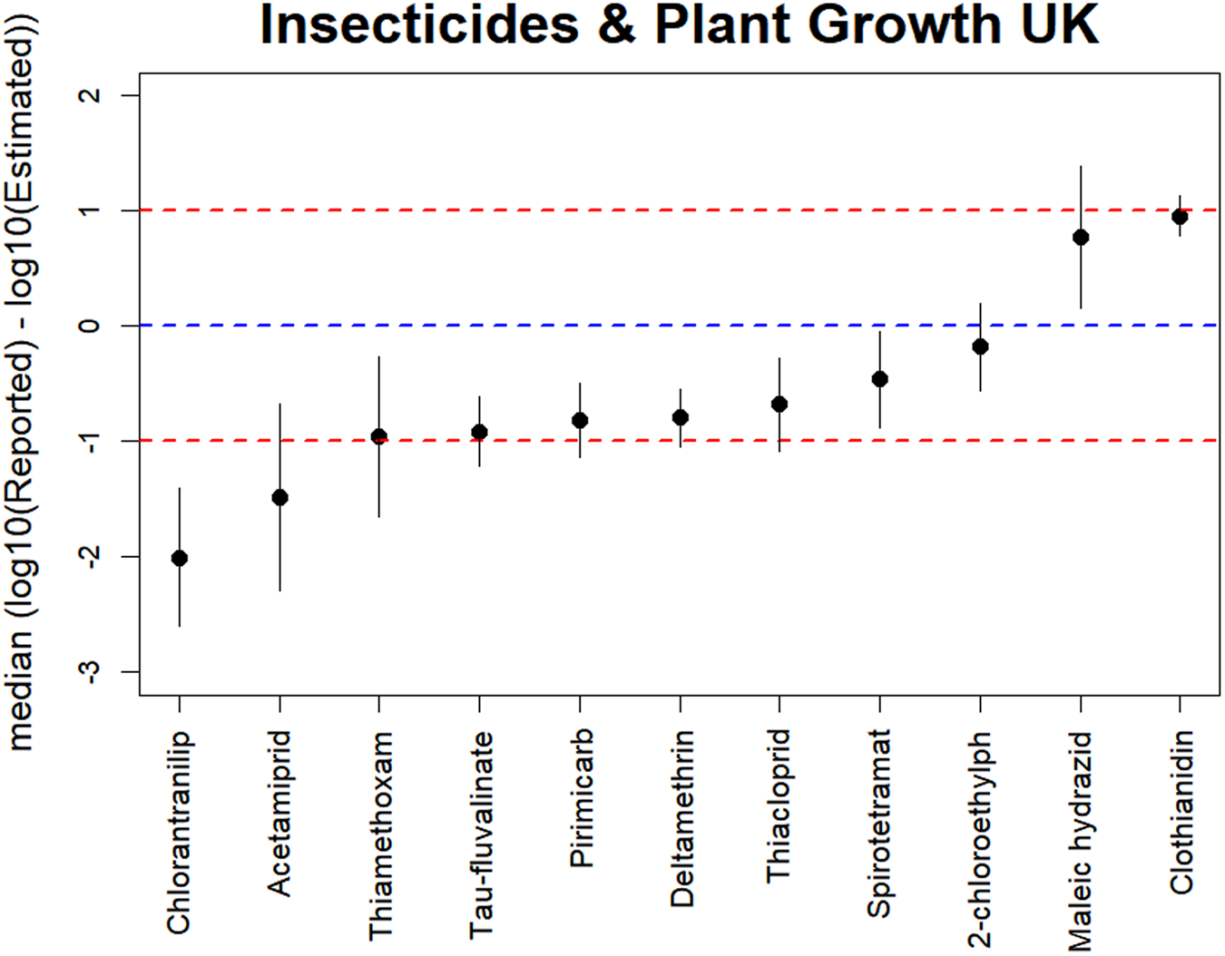
Vertical lines showing the comparison between the estimates and the reported UK emissions for 9 AS of the Insecticides category (left) and 2 AS for Plant Growth in 11 UK regions. The dot in each vertical line is the median of the difference between log10 reported values and log10. The vertical lines show the median absolute deviation, as measure of robust error in each estimation. The dashed blue horizontal line indicates 1:1 relation. The red dashed horizontal lines indicate the 1:10 and 10:1 relationship.

Comparing the reported and modelled values in the UK for the Insecticides and plant growth regulators categories (Fig. 8), the differences are slightly higher for insecticides, although only twice exceeding one order of magnitude. In any case, the differences for insecticides are quite acceptable considering that this is a category of PPPs that was more complex to model because most of them are quite crop-specific and information was rarely available in the 8 reference countries.

## Usage Notes

The estimated amounts of PPP AS used in each NUTS Level 3 region of the EU can be used as a proxy for more detailed data where these are not available. As such, our estimates can feed model-based assessments of pesticide fate and transport, toxicity and impacts. An example of this can be found in^13^. It should be stressed that our estimates are based on heterogeneous data reported by 8 EU Member States during 2011–2017. As such, the reports do not reflect a specific year. Therefore, our estimates should be considered as representative of an average or reference use of pesticides during this period^11^.

### Supplementary information

SupplementaryInformation

## Data Availability

The code used for the whole procedure, is written in the R programming language, and is available on GitHub (https://github.com/angeludias/PPP_emissions).
