# Supplementary material for: Emissions of pesticides in the European Union: a new regional-level dataset
Source: Sci Data. 2023 Dec 5;10:869. doi: 10.1038/s41597-023-02753-4 (PMC10698185; doi:10.1038/s41597-023-02753-4)
Supplement: Supplementary file 1 — SupplementaryInformation [file 41597_2023_2753_MOESM1_ESM.pdf]

Supplementary Material

1 Table of contents

Table SI 1: *Data availability*

Table SI 2: CLC codes (corresponding with crops) included in each main agricultural class according to EUROSTAT, into 6 main Corine Land Classes)

Table SI 3: Goodness of fit metrics, namely NRMSE (Normalized Root Mean Square Error) and Nash-Sutcliffe Efficiency (NSE), for models M1 through M6 with respect to five active substances (AS): Glyphosate, Tebuconazole, Dimethomorph, Thiacloprid, and Esfenvalerate. The values of the goodness of fit metrics have been derived based on logarithmically transformed reference and modeled values

Fig. SI 1: Boxplots by AS categories presenting the statistical distribution of model prediction quality based on the R2 (coefficient of determination) metric. The data reflect the results of the 10-fold cross-validation process for the best model in each AS applied to the arable class. For the SMAPE metric, a result closer to "1" indicates a better outcome.

Fig. SI 2: Boxplots by AS categories presenting the statistical distribution of model prediction quality based on the NRMSE (normalized root mean square error) metric. The data reflect the results of the 10-fold cross-validation process for the best model in each AS applied to the arable class. For the SMAPE metric, a result closer to zero indicates a better outcome.

Fig. SI 3: Boxplots by AS categories presenting the statistical distribution of model prediction quality based on the SMAPE (Symmetric Mean Absolute Percentage Error) metric. The data reflect the results of the 10-fold cross-validation process for the best model in each AS applied to the arable class. For the SMAPE metric, a result closer to zero indicates a better outcome.

Fig. SI 4: Boxplots by AS categories presenting the statistical distribution of model prediction quality based on the NMAE (normalized mean absolute error) metric. The data reflect the results of the 10-fold cross-validation process for the best model in each AS, applied to the arable class. For the NSE metric, a result closer to zero indicates a better outcome.

Fig. SI 5: Boxplots by AS categories presenting the statistical distribution of model prediction quality based on the NSE (Nash–Sutcliffe model efficiency coefficient) metric. The data reflect the results of the 10-fold cross-validation process for the best model in each AS, applied to the arable class. For the NSE metric, a result closer to one indicates a better outcome.

2 Spatial Resolution

NUTS (Nomenclature of Territorial Units for Statistics) is a geocode standard used by the European Union to divide up the territories of its member countries. The NUTS classification is hierarchical and divides countries into larger regions, smaller regions, and finally, local administrative units.
NUTS Level 3 refers to the third level in this hierarchy, which is the level at which relatively small regions are defined within a country. These regions are designed to be homogeneous with respect to certain socio-economic factors and are often used for statistical purposes.
NUTS level 3 units are used as a way of aggregating pesticide use data according to small geographic regions within European countries. This allows for a more detailed and nuanced analysis of pesticide use patterns than would be possible using large administrative units.
The average area of a NUTS level 3 unit can vary significantly between different European countries, as it depends on factors such as population density and geographic size.

3 Spatial model of emission data from selected countries

As already mentioned, one of the focal point of this analysis is to retrieve data on use/sales of individual pesticides for a number of European Countries at a reasonable spatial scale. Therefore, a workshop was organized with Experts from European Countries at the beginning of 2019 with this main objective (Galimberti, F., Dorati, C., Udias, A., Pistocchi, A., 2020), and the gathered data are listed in the following table.

Table SI 1: *Data availability*

Country	Spatial Resolution	Temporal Coverage	Data available at
Belgium	Country level	2011-2017	https://fytoweb.be/fr/plan-de-reduction/vigilance/donnees-de-vente
Denmark	Country level	2012	https://www2.mst.dk/Udgiv/publikationer/2014/12/978-87-93283-33-6.pdf
France	Province level	2010-2017	http://www.data.eaufrance.fr/jdd/bd45f801-45f7-4f8c-b128-a1af3ea2aa3e
Germany	Country level	2017	MS Excel Spreadsheets
Ireland	Country level	2011-2015*	http://www.pcs.agriculture.gov.ie/sud/pesticidestatistics/
Italy	Region level	2012	https://www.sian.it
The Netherlands	Country level	2012 and 2016	https://opendata.cbs.nl/statline/#/CBS/nl/dataset/84010NED/table?dl=1CE23
Spain	Province level	2013*	https://www.mapa.gob.es/es/estadistica/temas/estadisticas-agrarias/agricultura/estadisticas-medios-produccion/fitosanitarios.aspx

4 Data processing

A different Country-to-Country spatial and temporal variability of the data stands out immediately, so that a harmonizing process was necessary to create a unique dataset according to a robust template. In addition, some other technical aspects have been taken into account, such as: pesticide ids and names in the different languages; data provided on pesticide as real use vs sales data; data provided on active substances vs pesticide products; specifics on application on crops and crop application coverage (Country main peculiar crops vs all crops).

The provided data refer to different time trends and years. When possible, data have been harmonized using the average value in order to obtain a hypothetical average year of pesticide application.

Due to the spatial (referring NUTS) and crop variability in terms of different crop groups and different details on single crops, a specific analysis was performed to harmonize all the provided agricultural

information to a unique template: a combination of Eurostat NUTS2 agricultural dataset (https://appsso.eurostat.ec.europa.eu/nui/show.do?dataset=ef_lus_allcrops&lang=en) and Corine Land Cover NUTS3 was used as base spatial template (Galimberti, F., Dorati, C., Udias, A., Pistocchi, A., 2020).

Real uses and pesticide sales have been treated as similar data and managed accordingly. All the retrieved data were reduced to the pesticide active substance, so that where data were expressed as pesticide products, the effective kilograms were calculated according to the content percentage of active ingredient/s in the pesticide products. In the workshop, experts have also provided information on pesticide authorized uses on crops/ group of crops, so it was possible to spatialize the amount of pesticides on crop areas. National and Regional data of pesticide loads were spatialized according to the distribution percentage of crops within each NUTS3.

The resulting values were stored into dataset based on kg of each active substance, per specific crops, by NUTS3.

5 Crop codes in each Corine Land Classes

Table SI 2 shows which EUROSTAT crop categories are associated with each of the six land cover classes.

Table SI 2*: CLC codes (corresponding with crops) included in each main agricultural class according to EUROSTAT, into 6 main Corine Land Classes)*

Arable land	Fruit tree	Grassland	Vineyards	Olive groves	Rice fields
C1110, C1120, C1200, C1300, C1400, C1600_1700_1900, C1500, E0000, G1000, G2000, G3000, G9000, I1110, I12200, I12900, I3000, I4000, I5000, I9000, K0000, L0000, N0000S, N0000T, P9000, P1000, R2000, R1000, R9000, V0000_S0000S, V0000, S0000T, U1000, ARA99	F1000<br>F2000<br>F3000<br>F4000<br>T0000	J1000<br>J2000	W1000<br>W1200<br>Q0000	O1000	C2000

6 List of Databases on registered plant protection products in the EPPO (European and Mediterranean Plant Protection Organization) region

Most of the information (labels) concerning the amounts of each active substance in each commercial product in all member states was extracted from the following website.

https://www.eppo.int/ACTIVITIES/plant_protection_products/registered_products

An example of a product label for France could be the CAMIX, which can be viewed at the following address:

https://ephy.anses.fr/ppp/camix

CAMIX with an S-metolachlor content of 400 g/L product and also contains other active substances:

benoxacore : 20 g/L

mesotrione : 40 g/L

7 Metrics of model prediction quality from the 10-fold cross validation assessment

Table SI 3: *Goodness of fit metrics, namely NRMSE (Normalized Root Mean Square Error) and Nash-Sutcliffe Efficiency (NSE), for models M1 through M6 with respect to five active substances (AS): Glyphosate, Tebuconazole, Dimethomorph, Thiacloprid, and Esfenvalerate. The values of the goodness of fit metrics have been derived based on logarithmically transformed reference and modeled values.*

	Glyphosate		Tebuconazole		Dimethomorph		Thiacloprid		Esfenvalerate	
	NRMSE	NSE	NRMSE	NSE	NRMSE	NSE	NRMSE	NSE	NRMSE	NSE
M1	0.719	0.482	0.904	0.182	0.970	0.059	0.857	0.265	1.114	-0.242
M2	0.722	0.478	0.904	0.182	0.972	0.055	0.842	0.289	1.100	-0.212
M3	0.846	0.284	1.055	-0.115	0.795	0.367	0.882	0.221	0.895	0.197
M4	1.049	-0.10	1.085	-0.179	0.946	0.104	0.854	0.269	1.173	-0.378
M5	0.731	0.464	0.801	0.358	0.487	0.762	0.768	0.409	0.781	0.389
M6	0.572	0.672	0.663	0.560	0.972	0.055	0.800	0.359	1.100	-0.213

8 Statistical distribution of the result for the cross-validation process according to different metrics

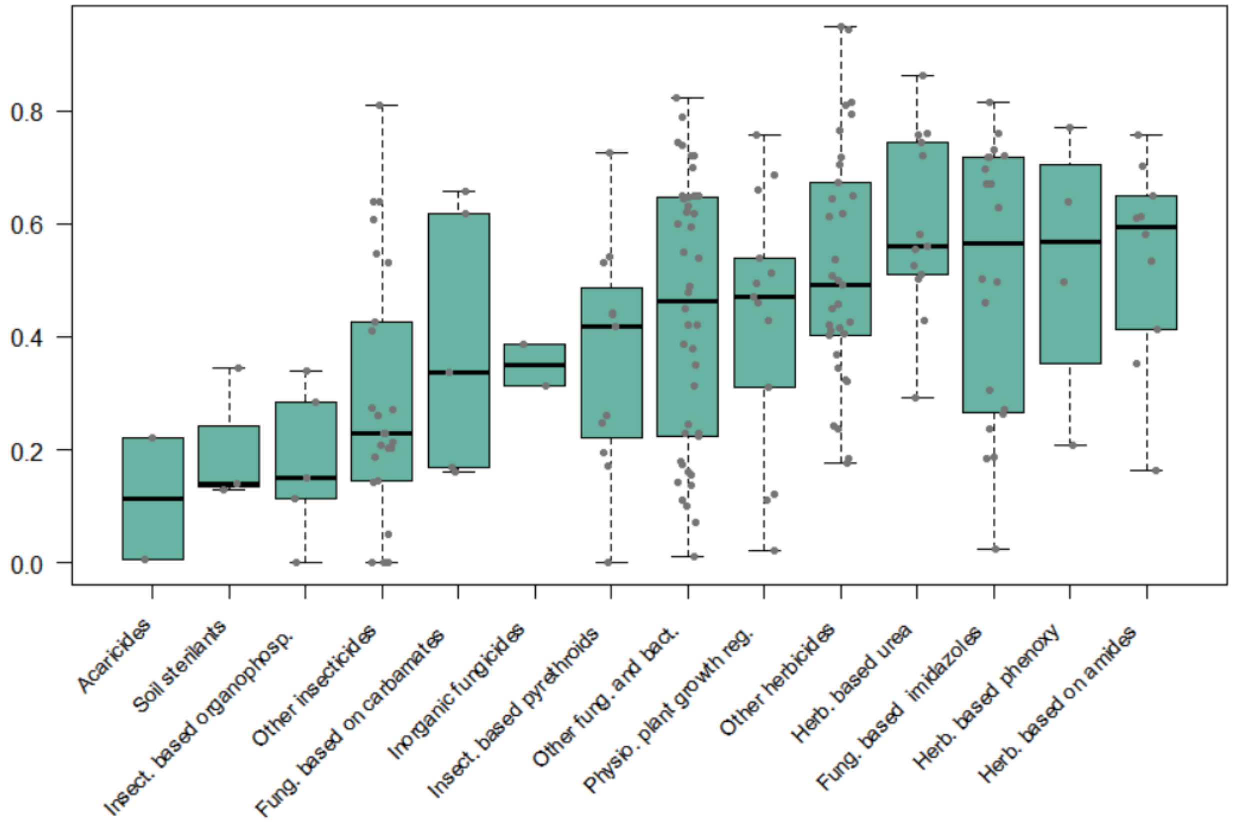

Fig. SI 1: *Boxplots by AS categories presenting the statistical distribution of model prediction quality based on the R2 (coefficient of determination) metric. The data reflect the results of the 10-fold cross-validation process for the best model in each AS applied to the arable class. For the SMAPE metric, a result closer to "1" indicates a better outcome.*

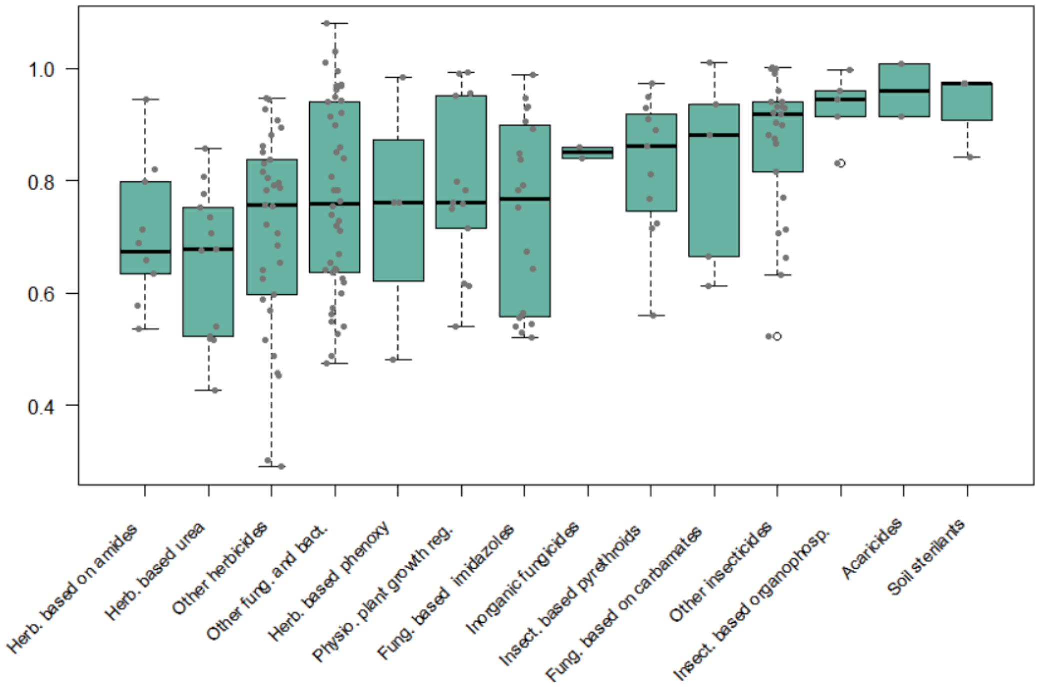

Fig. SI 2: *Boxplots by AS categories presenting the statistical distribution of model prediction quality based on the NRMSE (normalized root mean square error) metric. The data reflect the results of the 10-fold cross-validation process for the best model in each AS applied to the arable class. For the SMAPE metric, a result closer to zero indicates a better outcome.*

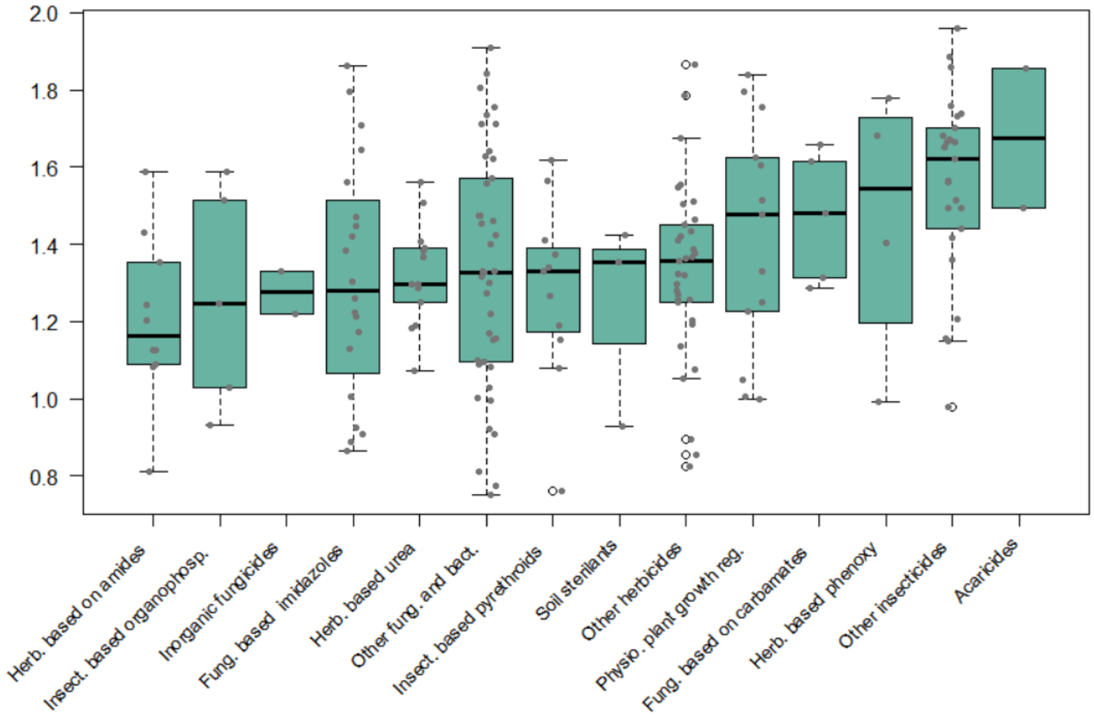

Fig. SI 3: *Boxplots by AS categories presenting the statistical distribution of model prediction quality based on the SMAPE (Symmetric Mean Absolute Percentage Error) metric. The data reflect the results of the 10-fold cross-validation process for the best model in each AS applied to the arable class. For the SMAPE metric, a result closer to zero indicates a better outcome.*

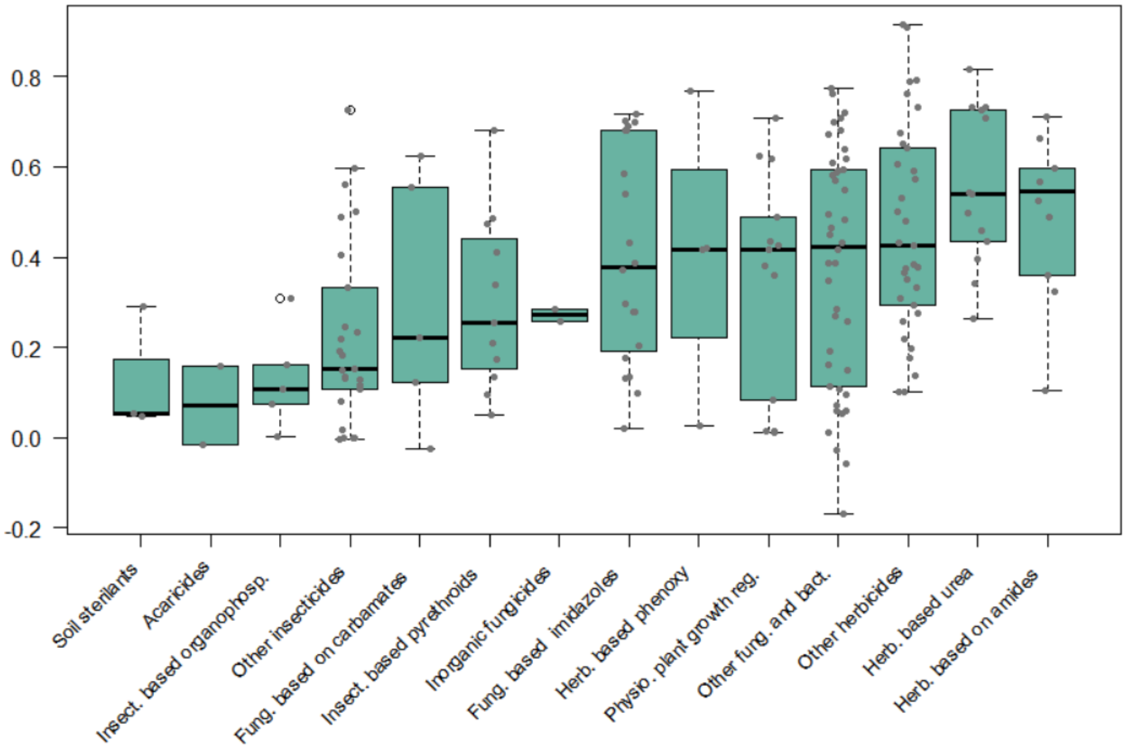

Fig. SI 4: *Boxplots by AS categories presenting the statistical distribution of model prediction quality based on the NMAE (normalized mean absolute error) metric. The data reflect the results of the 10-fold cross-validation process for the best model in each AS, applied to the arable class. For the NSE metric, a result closer to zero indicates a better outcome.*

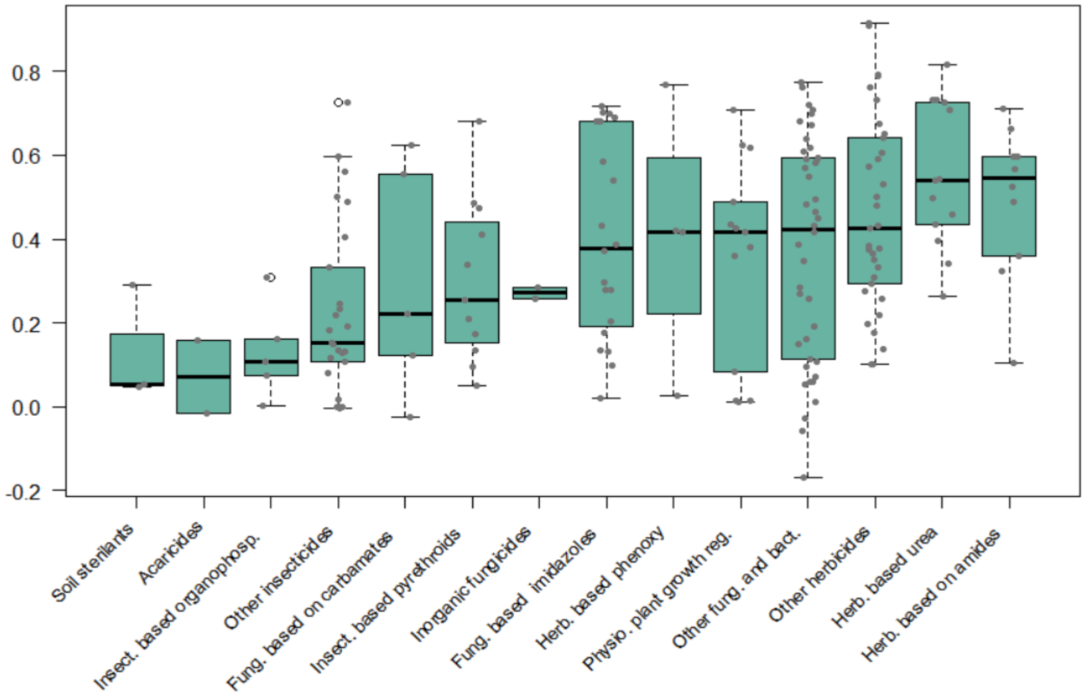

Fig. SI 5: *Boxplots by AS categories presenting the statistical distribution of model prediction quality based on the NSE (Nash–Sutcliffe model efficiency coefficient) metric. The data reflect the results of the 10-fold cross-validation process for the best model in each AS, applied to the arable class. For the NSE metric, a result closer to one indicates a better outcome.*
